# The correlation between dermoscopy and clinical and pathological tests in the evaluation of skin photoaging

**DOI:** 10.1111/srt.13578

**Published:** 2024-01-14

**Authors:** Jie Zhao, Xun Zhang, Qiao Tang, Yunfeng Bi, Limei Yuan, Binbin Yang, Mei Cai, Jianzhong Zhang, Danqi Deng, Wenting Cao

**Affiliations:** ^1^ Department of Dermatology The Second Affiliated Hospital of Kunming Medical University Kunming Yunnan China; ^2^ Department of Dermatology Qionglai City Medical Center Hospital Qionglai Sichuan China; ^3^ Department of Dermatology Peking University People's Hospital Beijing China

**Keywords:** clinical evaluation, correlation, dermoscope, histopathology, skin photoaging

## Abstract

**Background:**

There are no standards for evaluating skin photoaging. Dermoscopy is a non‐invasive detection method that might be useful for evaluating photoaging.

**Objective:**

To assess the correlation between the dermoscopic evaluation of photoaging and clinical and pathological evaluations.

**Methods:**

The age, clinical evaluation (Fitzpatrick classification, Glogau Photoaging Classification, and Chung's standardized image ruler), histopathology (Masson staining and MMP‐1 immunohistochemistry), and dermoscopy (Hu's and Isik's) of 40 donor skin samples were analyzed statistically, and Spearman rank correlation analysis was performed.

**Results:**

There was a robust correlation between the total Hu scores and Isik dermoscopy. The correlation of dermoscopy with histopathology was higher than that of clinical evaluation methods. There is a strong correlation between telangiectases and lentigo. Xerosis, superficial wrinkle, diffuse erythema, telangiectases, and reticular pigmentation were significantly correlated with the three clinical evaluation methods. Superficial wrinkles were correlated with Masson, MMP‐1, various clinical indicators, and other dermoscopic items.

**Conclusion:**

There is a good correlation between dermoscopy and clinical and histopathological examination. Dermoscopy might help evaluate skin photoaging.

## 1 INTRODUCTION

Skin photoaging is characterized by roughness, dryness, uneven color, and wrinkles caused by long‐term ultraviolet radiation on the skin.^1^ Photoaging is also a potential risk factor for various skin inflammatory, immune, and neoplastic diseases.^2^ A convenient, rapid, and accurate diagnostic method is critical to preventing and treating photoaging and related skin diseases. Photoaged skin can show changes in clinical appearance, histopathology, immunohistochemistry, and gene expression. However, there is currently no gold standard for photoaging diagnosis and severity evaluation. Although clinical evaluation methods are relatively convenient, their accuracy depends on practitioner experience, and there is substantial subjectivity; the results between evaluators lack comparability.^3^ Pathology and immunohistochemistry analyze the degree of histopathological changes in the skin and the expression levels of related molecules^4^, ^5^; however, because they are time‐consuming, invasive, and lack standardized quantitative standards, they are limited to research and unsuitable for clinical use.

Dermoscopy is a non‐invasive diagnostic technique used for detecting photoaging.^6^, ^7^ The Hu^8^ and Isik^9^ dermoscopy scores are currently used methods for evaluating photoaging. To explore the accuracy of dermoscopy in photoaging evaluation, we analyzed the correlation between dermoscopy scores and clinical evaluation methods, immunohistochemistry, Masson staining, and age in photoaging evaluation to determine whether dermoscopy can link clinical and histopathological aspects when applied to photoaging and promote dermoscopy for evaluating photoaging in skin.

## 2 MATERIALS AND METHODS

### 2.1 Donors

From January to June 2021, 40 patients underwent surgery in the Department of Dermatology, Burns, Orthopedics, and Plastic Surgery of the Second Affiliated Hospital of Kunming Medical University. Their skin samples were collected from the face, elbow extension, outer calf, armpit, abdomen, groin, or thigh. All donors provided informed written consent.

Donors who met any of the following criteria were excluded: visible skin lesions at the sampling site; history of local topical medication at the sampling site within the past 3 months; systematic use of retinoids, anti‐inflammatories, or immunosuppressants within the previous 6 months; history of hormone replacement therapy; history of drug abuse; systemic diseases such as diabetes, smoking, alcohol abuse. The Medical Ethics Committee of the Second Affiliated Hospital of Kunming Medical University approved the study (No.: Shen‐PJ‐2020‐127).

## 3 METHODS

### 3.1 Clinical and dermoscopic evaluation of photoaged skin

Before surgery, clinical and dermoscopic photographs (DermLite DL4 portable dermoscopy) were taken from the surgical sites, and the Fitzpatrick classifications of the donors were recorded.^10^ Clinical evaluation of photoaging was conducted using the Glogau Photoaging Classification^11^ and Chung's photographic scales for grading wrinkles and dyspigmentation.^12^ The dermoscopic evaluation methods of Hu^8^ and Isik^9^ were used to evaluate photoaging.

### 3.2 Pathological and immunohistochemical evaluation of photoaging skin

The entire layer of skin (0.5 cm^2^) was surgically removed, and the surgical site was more than 0.5 cm from the edge of the lesion. The skin tissue was fixed in 10 mL 4% paraformaldehyde phosphate buffer for 24 h, embedded in paraffin, and sliced, followed by matrix metalloproteinases‐1^13^ (MMP‐1, Proteintech, 10371‐2‐AP), immunohistochemistry, and Masson staining^14^ (Solarbio, G1340) according to the manufacturer's instructions. The results of MMP‐1 and Masson were analyzed by positive area percentage (positive area/tissue area).^15^

### 3.3 Statistical analysis

Statistical analysis was performed using SPSS 26.0 and R 4.3.0. For continuous variables with normal distribution, mean ± standard deviation was used. For continuous variables with non‐normal distribution, median and quartile were used. Categorical variables were expressed as a rate (%) or proportion (%). Correlation analysis was performed using Spearman rank correlation analysis, and *p* < 0.05 defined statistical significance.

## 4 RESULTS

### 4.1 Clinical and pathological donor data

**FIGURE 1:**
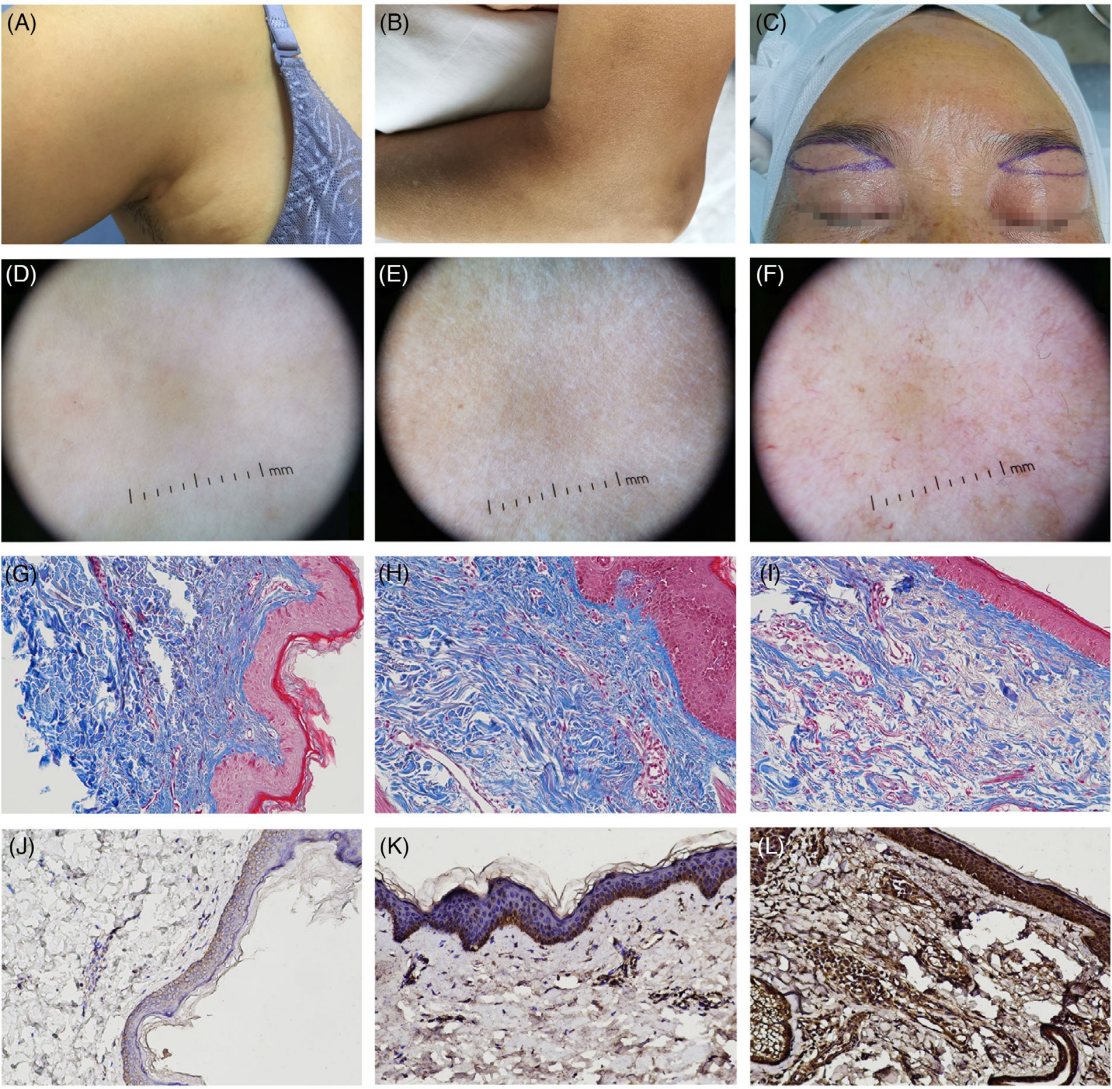
Typical clinical, dermoscopic, and histopathological photos of the donors. (A) A clinical photo of the axillary skin of a 51 years old woman. (B) A clinical photo of the left upper arm of a 56 years old woman. (C) A clinical photo of a 48 years old woman under the eyebow. (A)–(F) are dermoscopic photos of (A)–(C) (×10). (D) Mild xerosis. (E) Moderate xerosis, reticular pigmentation, homogeneous pigmentation in a patchy distribution, superficial wrinkles. (F) Mild xerosis, homogeneous pigmentation in a patchy distribution, linear vessels, branching vessels, lentigo, diffuse erythema, telangiectases. (G)–(I) are the Masson staining results of (A)–(C)(×200). (J)–(L) are the MMP‐1 immunohistochemical results of (A)–(C)(×200).

The 40 donors included 25 males and 15 females. The general information, Fitzpatrick classification, clinical grading of photoaging (Figure 1A–C), pathology, and immunohistochemistry (Figure 1G–L) of the donors are displayed in Tables 1 and 2. For the convenience of statistical analysis, clinical grades I to IV were assigned 1−4 points, and grades 1−5 were assigned 1−5. Donors with a wrinkle rating of 5−7 were 0 in both groups, while donors with a pigment rating of 0 were 0 in both groups and were omitted from Table 1. Age and histopathology indicators were not normally distributed, and the median and quartile were used for statistical description (Table 2).

**TABLE 1:** Clinical data of donors (%).

Item	Class	*N* (%)
Fitzpatrick scale	I	1 (2.5)
	II	13 (32.5)
	III	19 (47.5)
	IV	7 (17.5)
Glogau scale	I	15 (37.5)
	II	18 (45.0)
	III	5 (12.5)
	IV	2 (5.0)
Standardized image ruler wrinkle	0	4 (10.0)
	1	9 (22.5)
	2	14 (35.0)
	3	5 (12.5)
	4	8 (20.0)
Standardized image ruler pigment	1	7 (17.50)
	2	9 (22.5)
	3	12 (30.0)
	4	10 (25.0)
	5	2 (5.0)

**TABLE 2:** Age, pathology, and immunohistochemical results.

	25th quartile	50th quartile	75th quartile
Age	28.00	43.00	52.00
Masson	40.61	51.14	55.75
MMP‐1	121.63	169.23	182.53

### 4.2 Dermoscopic characteristics of the donors

The dermoscopic features evaluated by Hu and Isik scores are displayed in Table 3 (Figure 1D–F). The positive dermoscopic manifestation is given one point, and the negative is given zero points. Due to the presence of telangiectases in Hu and Isik dermoscopic scores, to evaluate the impact of repeated scoring of it on the results, we included “Hu+Isik‐telangiectases.” The total scores of the four dermoscopes were not normally distributed and were statistically described by the median and quartile.

**TABLE 3:** Hu, Isik dermoscopic features of donors (%).

Hu's dermoscopic characteristics		*N* (%)	
Xerosis	Mild	28 (72.5)	
	Moderate	6 (12.5)	
	Severe	0 (0)	
Uneven pigmentation	Small brown globules	8 (17.5)	
	Reticular pigmentation	21 (50)	
	Homogeneous pigmentation in a patchy distribution	23 (55)	
Vascular telangiectasia	Linear vessels	9 (20)	
	Branching vessels	5 (15)	
Isik's dermoscopic characteristics		*n* (%)	
Actinic keratosis		0 (0)	
Lentigo		9 (25)	
Diffuse erythema		24 (57.5)	
Hypo‐hyperpigmented macules		21 (55)	
Yellowish discoloration		7 (17.5)	
White line		6 (15)	
Telangiectasias		8 (17.5)	
Superficial wrinkles		9 (20)	
	25th quartile	50th quartile	75th quartile
Hu's total score	2	2	4
Isik's total score	4	6	9
Hu + Isik	4	6	10
Hu + Isik‐ telangiectasias	4	6	10

### 4.3 Correlation analysis

**FIGURE 2:**
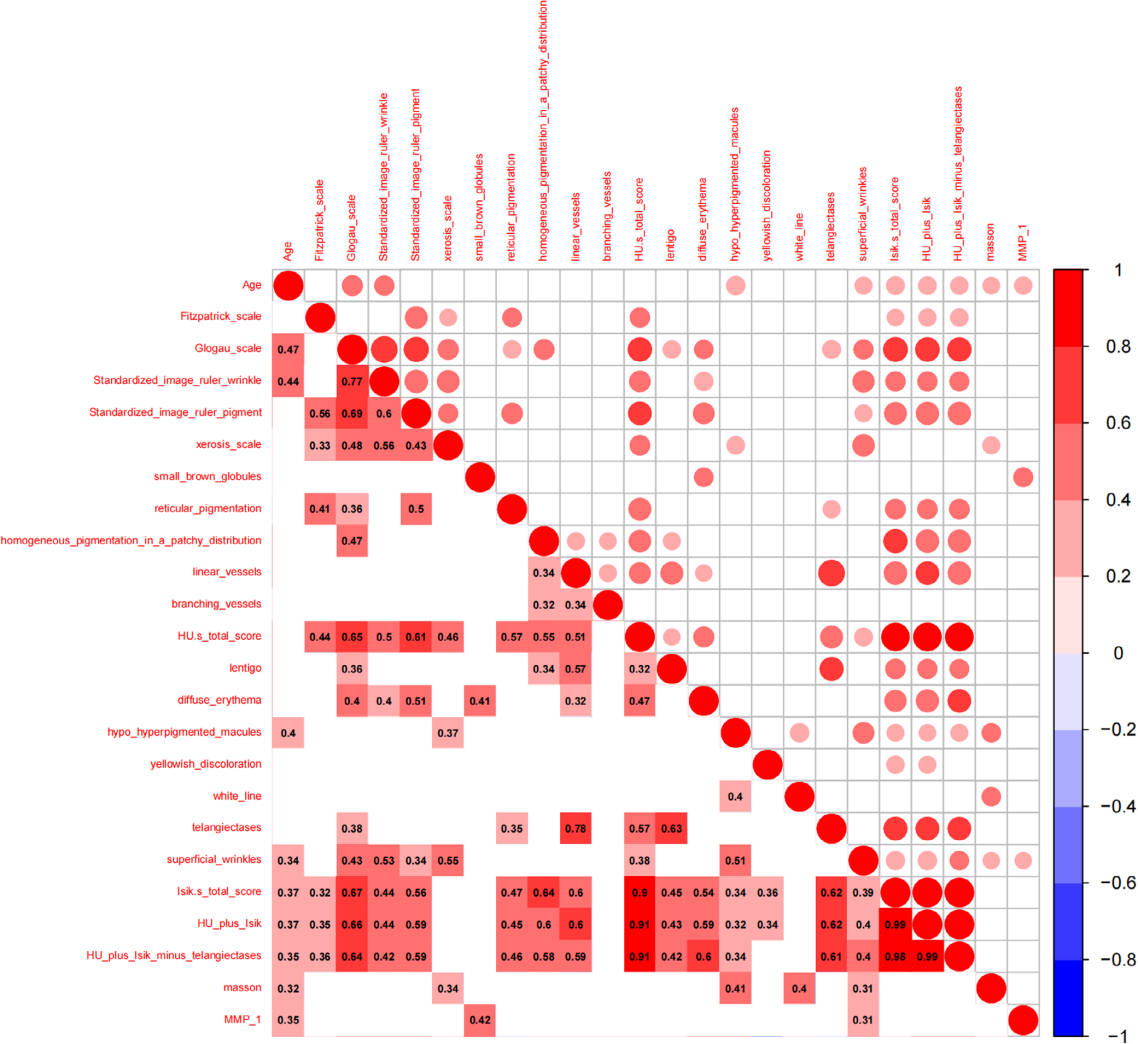
Correlation analysis between age, dermoscopy, clinical grading, and histopathology: the size of the ball in the upper right part of the figure represents the size of the *r*‐value, and the lower left part of the figure represents the corresponding *r*‐value; Red represents positive correlation, blue represents negative correlation; The darker the color, the stronger the correlation. The *r*‐values in the figure are statistically significant (the absolute value of *r* is ≥0.3, and *p* < 0.05).

Spearman rank correlation analysis was performed among age, dermoscopic score, clinical grading score, and histopathological results (Figure 2).

## 5 DISCUSSION

Skin aging can be caused by external factors such as ultraviolet radiation, smoking, and exposure to harmful chemicals. Ultraviolet radiation has the most potent effect on the exogenous factors leading to skin aging, and aging caused by ultraviolet radiation is called photoaging.^16^ Compared with endogenous aging, photoaging skin results in relaxation, significant dryness, and peeling. There are also unique manifestations of photoaging, such as skin thickening, excessive keratinization, pigmentation, uneven pigmentation, erythema, and telangiectasia.^17^, ^18^

Dermoscopy is a non‐invasive image analysis technology that uses polarized or unpolarized light to visualize pigmentation patterns, vascular structures, and other epidermis and dermis morphological features that the naked eye cannot detect.^19^ Hu et al. classified the xerosis of photoaging skin observed using dermoscopy as mild, moderate, and severe.^8^ Uneven pigmentation was described as small brown globules, reticular pigmentation, or homogeneous pigmentation in a patchy distribution. Vascular telangiectasia was classified as linear and branching vessels. Isik et al. described 12 types of dermoscopic manifestations of photoaging, including diffuse erythema, hyperpigmented macules, yellow discoloration, white line, telangiectases, and super wrinkles, and developed a dermoscopic photoaging scale (DPAS).^9^ Compared with Hu's scale, Isik's scale is more diverse. Respati et al. correlated DPAS with sociodemographic characteristics (age, sex, skin type, smoking habits) and sun index scores and found that cheek preference, male, active smoking, Fitzpatrick type IV skin, and increased age had higher DPAS scores.^20^ There was no correlation between DPAS scores and the sun index. There were some similarities and overlaps between Hu's and Isik's dermoscopy photoaging evaluation, including linear vessels, branching vessels, and telangiectases; however, there are no studies of the correlation between Hu's and Isik's methods.

We found a robust positive correlation between the methods’ total scores (*r*‐values greater than 0.9). The repeated scoring of telangiectases had little effect on the correlation between the total score of Hu's plus Isik's and other items. The items were less correlated except for linear vessels and telangiectases, suggesting that they are independent. There was a moderate correlation between telangiectases and lentigo (*p* = 0.63). Although these manifestations originate from different histopathological foundations, their high correlation suggests that they may share a common or related mechanism of occurrence, which merits further research.

Age is an essential factor in accelerating skin aging.^21^ In the absence of precise criteria for evaluating photoaging, age affects photoaging severity. In our correlation analysis, age had a variable correlation with clinical evaluation and histopathology. There was a weak correlation between Isik's total score, hyperpigmented macules, super wrinkles, and age (*r* = 0.37, 0.4, and 0.34, respectively). However, there was no correlation between Hu's total score and terms under Hu's dermoscopy and age. This finding suggests that Isik's score correlates more with age than Hu's score.

Glogau's scale and Chung's photographic scales for grading wrinkles and dyspigmentation^12^ were applied for clinical evaluation. These scales focus on pigmentation and wrinkles to evaluate photoaging visually. Correlation analysis revealed a strong or moderate correlation among the three scales. The accuracy of these methods is substantially affected by the subjective experience of clinicians and the visibility of skin aging.

In dermoscopic scores, wrinkles and pigmentation are the primary features of photoaging. The advantage of dermoscopy is that it visualizes and refines pigmentation and wrinkles, substantially reducing subjectivity's influence. Furthermore, dermoscopy is conducive to earlier detection of slight pigmentation and wrinkles not readily recognizable to the naked eye. We observed high correlations between dermoscopic total scores and clinical scales. Xerosis, superior wrinkles, diffuse erythema, telangiectasis, and reticular pigmentation were significantly correlated with the three clinical scales.

Histopathology is a bridge connecting clinical and pathological mechanisms and genes. Masson staining can reveal the distribution of epidermal structure and dermal fibers. The photoaged epidermis shows irregular thickening or thinning; the epidermis is flat, the collagen fibers in the dermis are reduced, and the vascular network is disordered, curved, and dilated. Immunohistochemistry can detect MMP expression, which leads to collagen destruction, reduction of dermal collagen fibers,^17^ wrinkle formation, increased reticular fibers, and elastic fiber degeneration.^22^ The accuracy of the pathological evaluation is better than the clinical scales; however, it is invasive, takes a long time, and is difficult to use.

Khan et al. used collagen density to quantify photoaged skin in mice before and after treatment.^5^ Sachs et al. used elastic proliferation to evaluate the degree of skin photoaging.^23^ Hughes et al. commented that elastic hyperplasia of skin tissue is the most common evaluation index of photoaging and analyzed the correlation with epidermal thickness, p53 positive cell ratio, Masson staining, and other histopathological tests.^24^ The histopathological tests of skin photoaging vary, and there is no quantitative standard. Until now, a correlation analysis between histopathology and age, clinical evaluation, dermoscopy, and other tests has not been reported.

In the present study, we found a correlation between Masson staining and MMP‐1 immunohistochemistry detection and age, which partially confirms that pathological indicators and age can reflect the severity of skin photoaging. The Masson staining positively correlated with the Glogau scale and dermoscopic features. The correlations between the four dermoscopic features of hypo/hyperpigmented macules (*r* = 0.41), white lines (*r* = 0.4), xerosis scale (*r* = 0.34), superior wrinkles (*r* = 0.31), and Masson were more significant than that of Glogau scale (*r* = 0.32), supporting the advantages of dermoscopic evaluation of photoaging.

In the correlation analysis of MMP‐1, it was only correlated with two dermoscopic terms: small brown globules (*r* = 0.42) and superficial wrinkles (*r* = 0.31). MMP‐1 had no significant correlation with clinical scales, suggesting that dermoscopy and histopathology, which are invasive but closer to gene and pathogenesis detection, correlate better than clinical evaluation. Superior wrinkles correlated with Masson, MMP‐1, various clinical scales, and other dermoscopy terms. Further research should determine whether superior wrinkles can increase its weight in the photoaging score of dermoscopy.

Our samples were taken from exposed (such as the face, elbow, and outer calf) and non‐exposed areas (such as thighs, abdomen, and armpits). Despite the so‐called non‐exposed parts, there were individual differences and almost no completely non‐exposed samples. Furthermore, the characteristic pathological manifestations of actinic keratoses are such that they might significantly impact the Masson and MMP‐1 tests. Therefore, we excluded skin appearing to contain actinic keratosis as the sampling site; this decision resulted in zero observations of this phenomenon on Isik's score. Because the testing was invasive, there were relatively willing donors. Finally, there is no gold standard for diagnosing skin photoaging, and our data types are diverse, which limits the selection of statistical methods. The testing efficiency of Spearman rank correlation analysis is lower than that of Pearson correlation analysis, which may be one of the reasons for the generally low correlation coefficients.

Dermoscopy serves as a bridge between clinical and histopathological tests. It can reveal the microscopic structure from the epidermis to the superficial layer of the dermis invisibly and dynamically.^25^ We found that the total Hu and Isik dermoscopy scores were highly correlated, and their correlation with histopathology was higher than that of clinical scales. These findings suggest that dermoscopy can evaluate skin photoaging. However, some dermoscopic terms were not strongly correlated with clinical and histopathological features; therefore, they can be simplified when formulating new dermoscopic photoaging evaluation criteria in the future.

## CONFLICT OF INTEREST STATEMENT

The authors have no conflict of interest to declare.

## ETHICS STATEMENT

The patients in this manuscript have given written informed consent to publication of their case details. The Medical Ethics Committee of the Second Affiliated Hospital of Kunming Medical University approved the study (No.: Shen‐PJ‐2020‐127).

## Data Availability

The data that support the findings of this study are available from the corresponding author upon reasonable request.
